# Targeted dianthin is a powerful toxin to treat pancreatic carcinoma when applied in combination with the glycosylated triterpene SO1861

**DOI:** 10.1002/1878-0261.12115

**Published:** 2017-09-15

**Authors:** Cheenu Bhargava, Horst Dürkop, Xiangli Zhao, Alexander Weng, Matthias F. Melzig, Hendrik Fuchs

**Affiliations:** ^1^ Institute for Laboratory Medicine Clinical Chemistry and Pathobiochemistry Charité – Universitätsmedizin Berlin Germany; ^2^ Pathodiagnostik Berlin Germany; ^3^ Institute for Pharmacy Freie Universität Berlin Germany

**Keywords:** endosomal escape, epidermal growth factor receptor, pancreatic carcinoma, SO1861, targeted toxin, xenograft

## Abstract

Targeted cancer therapy provides the basis for the arrest of tumor growth in aggressive pancreatic carcinoma; however, a number of protein‐based targeted toxins lack efficacy due to insufficient endosomal escape after being endocytosed. Therefore, we tested a fusion protein of the ribosome‐inactivating protein dianthin and human epidermal growth factor in combination with a glycosylated triterpene (SO1861) that serves as an endosomal escape enhancer. *In vitro* investigations with the pancreatic carcinoma cell lines BxPC‐3 and MIA PaCa‐2 revealed no significant differences to off‐target cells in the half maximal inhibitory concentration (IC_50_) for the fusion protein. In contrast, combination with SO1861 decreased the IC_50_ for BxPC‐3 cells from 100 to 0.17 nm, whereas control cells remained unaffected. Monotherapy of BxPC‐3 xenografts in CD‐1 nude mice led to a 51.7% average reduction in tumor size (40.8 mm^3^) when compared to placebo; however, combined treatment with SO1861 resulted in a more than 13‐fold better efficacy (3.0 mm^3^ average tumor size) with complete regression in 80% of cases. Immunohistochemical analyses showed that tumor cells with lower target receptor expression are, in contrast to the combination therapy, able to escape from the monotherapy, which finally results in tumor growth. At the effective concentration, we did not observe liver toxicity and saw no other side effects with the exception of a reversible skin hardening at the SO1861 injection site, alongside an increase in platelet counts, plateletcrit, and platelet distribution width. In conclusion, combining a targeted toxin with SO1861 is proven to be a very promising approach for pancreatic cancer treatment.

**Abbreviation**
DMEM: Dulbecco's modified Eagle's medium
EGF: epidermal growth factor
EGFR: epidermal growth factor receptor
HE: hematoxylin–eosin
IC_50_: concentration that inhibits cell survival to half maximal extent
KRAS: Kirsten rat sarcoma viral oncogene homolog
MTT: 3‐(4,5‐dimethylthiazol‐2‐yl)‐2,5‐diphenyltetrazolium bromide
Ni‐NTA: nickel nitrilotriacetic acid
PBS: phosphate‐buffered saline
RPMI medium: Roswell Park Memorial Institute medium
SDS/PAGE: sodium dodecyl sulfate/polyacrylamide gel electrophoresis
SO1861: an amphipathic glycoside of 1861 Da isolated from *Saponaria officinalis*

## 1 Introduction

Pancreatic ductal adenocarcinoma is the fourth most common cause of death from cancer in the USA; it was estimated that 48 960 new cases would be diagnosed and that 40 560 patients would succumb to the disease over the course of 2015 (Siegel *et al*., 2015). The identification of various molecular pathways that are very prominent in pancreatic carcinoma has contributed to the development of targeted therapies including monoclonal antibodies and small‐molecule inhibitors (Jones *et al*., 2008). In patients with advanced‐stage disease, modest improvements in survival have recently been attained with the chemotherapy regimen FOLFIRINOX (folinic acid, 5‐fluorouracil, irinotecan, and oxaliplatin) or albumin‐bound paclitaxel (nab‐paclitaxel) plus gemcitabine chemotherapy (Conroy *et al*., 2011; Von Hoff *et al*., 2013). The combination of gemcitabine and the epidermal growth factor receptor (EGFR; HER1; erbB1) tyrosine kinase inhibitor erlotinib gained regulatory approval following a 12‐day improvement in median survival compared with gemcitabine alone in a large, randomized phase III trial (Moore *et al*., 2007). A factor limiting the efficacy of pancreatic cancer treatment is impaired drug delivery as a result of the unique desmoplastic response – the pervasive growth of dense fibrous tissue around the tumor – that occurs in pancreatic ductal adenocarcinoma (Garrido‐Laguna and Hidalgo, 2015).

The EGFR is overexpressed in a variety of human tumors including pancreatic, non‐small‐cell lung cancer, breast, head and neck, gastric, colorectal, esophageal, prostate, bladder, renal and ovarian cancers (Salomon *et al*., 1995). Overexpression of EGFR and its ligands is frequently observed in pancreatic cancer and correlates with poor prognosis, disease progression, and metastasis (Oliveira‐Cunha *et al*., 2011; Xie and Xie, 2015), supporting the prominent role of the EGFR family for malignant transformation, prevention of apoptosis, and drug resistance. The pancreatic islet cell is considered by some to be a signaling hub (Barker *et al*., 2013). Although the Kirsten rat sarcoma viral oncogene homolog (KRAS) is mutated and activated in > 90% of pancreatic cancers, EGFR expression has also been shown to be essential for KRAS‐driven pancreatic ductal adenocarcinoma (Eser *et al*., 2014; Morris *et al*., 2010).

Treatment with monoclonal antibodies has also gained importance in past decades. The anticancer effect of monoclonal antibodies is thought to be primarily mediated via antibody‐dependent cell cytotoxicity, although complement‐mediated lysis may also contribute. In addition to antibody‐dependent cell cytotoxicity, these antibodies can be linked to toxins resulting in direct killing of the tumor cell after internalization or linked to a radioisotope and used as radiopharmaceuticals (Dodson *et al*., 2011).

Several clinical trials examining EGFR tyrosine kinase inhibitors are under way. A recent phase 3 trial of EGFR inhibitors has shown to be ineffective, including the use of the monoclonal antibody cetuximab in patients with late‐stage pancreatic cancer. Nevertheless, the results of clinical trials that target EGFR seem to be promising because monotherapy with panitumumab provided significant clinical benefit in heavily pretreated patients without acquired resistance to prior cetuximab‐based regimens (Pietrantonio *et al*., 2013), while the clinical relevance and cost‐effectiveness remain debatable.

The first generation of targeted toxins, developed 35 years ago, employed chemical conjugates of antibodies with intact toxins, or toxins with attenuated cell‐binding properties. Although they showed tumor regression in some patients with lymphoma, they were typically ineffective because the constructs were heterogeneous, nonspecific, and were too large to infiltrate solid tumors (Ghetie and Vitetta, 2001). Recombinant DNA techniques were applied in the production of third‐generation targeted toxins to promote tumor specificity and penetration, and to reduce the cost and complexity of production. The cell‐binding domain of the toxin is genetically removed and the modified toxin fused with a ligand or with DNA elements encoding the Fv portion of an antibody in these constructs. A variety of toxins, mainly from plants, fungi, or bacteria, have been characterized, structurally optimized for *in vitro* stability, activity, and safety, and evaluated *in vivo* in animal studies and clinical trials. Among them, ricin, *Pseudomonas* exotoxin, and diphtheria toxin are the most frequently used. However, the development of targeted toxins for the treatment of solid tumors poses challenges such as the generation of an immune response against the toxin moiety, poor tumor penetration, and reduced half‐life. Targeted toxins with RNases as the cytotoxic moiety are recent examples aimed at reducing the immunogenicity (Chang *et al*., 2010; Huhn *et al*., 2001).

Cytosolic drug delivery is one of the major routes in cancer treatment (Yu *et al*., 2016). The toxins naturally consist of several domains—the cell‐binding or cell recognition domain, the translocation domain, which enables release of the toxin into the cytosol, and the catalytic domain responsible for cytotoxicity. In the development of targeted toxins, the binding domain of these toxins is replaced by cancer cell‐specific ligands. The cancer cell‐specific ligands direct the internalization of the toxins via receptor‐mediated endocytosis; however, in many constellations, the toxin remains ineffective as it is recycled back to the cell surface or transported to the lysosomes where it is degraded (Fuchs *et al*., 2016). This might be the reason why no antibody‐targeted protein toxin has been approved for tumor therapeutic applications by the authorities to date. To overcome the insufficient endosomal escape, many strategies have been developed to weaken the membrane integrity of endosomal membranes by molecules that interact more or less directly with membranes (Fuchs *et al*., 2016). A common feature of these substances is that they are *per se* not target specific and distribute inside organisms with other kinetics than the targeted toxins. It is therefore obvious that the combination of these endosomal escape enhancers and targeted toxins can only be sufficiently tested *in vivo*; however, most of the published enhancers were only tested *in vitro*, which may explain lacking success of these strategies in clinical applications.

The aim of this study was to analyze the role of a targeted toxin consisting of the ribosome‐inactivating plant toxin dianthin and human epidermal growth factor (^His^Dianthin‐EGF) in combination with an amphipathic glycoside (SO1861) that serves as an endosomal escape enhancer in order to increase the cytotoxicity in EGFR‐overexpressing pancreatic carcinoma cells. A xenograft model was developed to determine the therapeutic efficacy of the targeted toxin alone and the targeted toxin in combination with SO1861. As amphipathic glycosides are known to have the potential to induce hemolysis, one focus of side effect examination was laid on complete blood count analysis.

## 2 Materials and methods

### 2.1 Expression and purification of recombinant proteins

Plasmid DNA (^His^Dianthin‐EGF‐pET11d) (Weng *et al*., 2012) was transformed into chemically competent *Escherichia coli* NiCo21 (DE3) (New England Biolabs^®^, Inc., Frankfurt am Main, Germany) and grown in 3 mL lysogeny broth medium supplemented with 50 μg·mL^−1^ ampicillin at 37 °C for 5 h at 200 rpm. These bacteria were used to inoculate 500 mL lysogeny broth supplemented with 50 μg·mL^−1^ ampicillin for overnight culture at 37 °C. Subsequently, the culture volume was scaled up to 2 L and bacteria were grown until an optical density (A_600_) of 0.9. Expression was induced by the addition of isopropyl β‐d‐1‐thiogalactopyranoside at a final concentration of 1 mm. Cells were further grown for 3 h at 37 °C and 200 rpm. After centrifugation (5 min, 5000 ***g***, 4 °C), cell pellets were resuspended in 20 mL phosphate‐buffered saline (Dulbecco's phosphate‐buffered saline (PBS) with Ca^2+^ and Mg^2+^, pH 7.4) and stored at −20 °C. After thawing, ^His^Dianthin‐EGF was released by ultrasound device (Branson Sonifier 250, G. Heinemann). The solution was centrifuged (30 min, 4 °C) and adjusted to 20 mm imidazole concentration. The construct contained an N‐terminal His‐tag and was purified by nickel nitrilotriacetic acid chromatography (Ni‐NTA Agarose; Qiagen, Hilden, Germany). After elution with imidazole (20–250 mm), the eluates were analyzed by sodium dodecyl sulfate/polyacrylamide gel electrophoresis (SDS/PAGE) (12%). Fractions containing ^His^Dianthin‐EGF were dialyzed against 2 L chitin binding domain buffer (20 mm tris(hydroxymethyl)‐aminomethane/HCl, 500 mm NaCl, 1 mm EDTA, 0.1% Tween‐20, pH 8.0) at 4 °C. Further purification by chitin column affinity chromatography served to remove bacterial proteins with binding activity for Ni‐NTA agarose. After elution with chitin binding domain buffer, the fractions were analyzed by SDS/PAGE (12%). Fractions containing ^His^Dianthin‐EGF were dialyzed against 5 L PBS at 4 °C. His‐tagged proteins were concentrated by Amicon centrifugal filter devices (10 kDa; Millipore, Eschborn, Germany). The protein concentration was determined by a bicinchoninic acid assay (Pierce, Rockford, IL, USA).

### 2.2 Isolation of SO1861

SO1861 was isolated from *Saponaria officinalis* L. Dried roots were finely ground and extracted by 90% methanol. The methanol was evaporated by vacuum distillation and cold acetone was added to the remaining aqueous extract. The resulting suspension was centrifuged and the pellet was dissolved in 20% methanol to 20 mg·mL^−1^ raw material. SO1861 was isolated by semipreparative high‐performance liquid chromatography using an UltraSep ES PHARM RP18E (7 μm, 250 × 8 mm) column from SepServ (Berlin, Germany) and a methanol/water (0.01% trifluoroacetic acid) gradient starting with 20% methanol to 80% methanol over 80 min. Flow rate was 1.5 mL·min^−1^. Fractions of SO1861 were freeze‐dried and analyzed by ESI‐MS. The isolated SO1861 was subjected to high‐performance thin‐layer chromatography analysis using corresponding silica gel 60 F254 plates (Merck Chemicals GmbH, Darmstadt, Germany) and the upper phase of a glacial acetic acid/water/butanol (2 : 8 : 10) mixture as solvent. Derivatization was performed by sulfuric acid (10%) and plates were dried at 140 °C. Purity of SO1861 was assessed by densitometry using the CAMAG TLC 4 Scanner (CAMAG, Berlin, Germany). Absorption was measured at 600 nm. Scans are shown in the Supporting information (Fig. S4).

### 2.3 Determination of N‐glycosylase enzymatic activity

The N‐glycosylase activity of ^His^Dianthin‐EGF was determined by an adenine release assay where adenine residues were released from herring sperm DNA (Weng *et al*., 2012). Briefly, 600 nm of the targeted toxin or free toxin was mixed with 10 μL (100 μg) herring sperm DNA and the volume made up to 100 μL by acetate buffer (50 mm sodium acetate, 100 mm KCl, pH 4.0). The mixture was incubated for 1 h in a shaker at 50 °C. Controls were either incubated with 600 nm bovine serum albumin or only with acetate buffer and herring sperm DNA. After separation of released adenine and herring sperm DNA by centrifugation with filter membranes (molecular mass cutoff 3 kDa, 5000 ***g***, 10 °C, 20 min; Sigma Aldrich, Hamburg, Germany), the absorbance of the flow‐through was measured at 260 nm by a Nano Drop spectrometer (ND‐1000; Peqlab Biotechnologie GmbH, Erlangen, Germany). Adenine release was calculated by the use of an adenine standard curve.

### 2.4 Cell culture

The human pancreatic carcinoma cell lines BxPC‐3 and MIA PaCa‐2 were cultured in Roswell Park Memorial Institute (RPMI) 1640 medium and Dulbecco's modified Eagle's medium (DMEM), respectively. NIH‐3T3 mouse embryonic fibroblast cells were cultured in DMEM. Medium was supplemented with 10% fetal bovine serum (BioChrom KG, Berlin, Germany), 100 U·mL^−1^ penicillin, and 100 μg·mL^−1^ streptomycin (Merck Chemicals GmbH). Cells were cultured at 37 °C and 5% CO_2_.

#### 2.4.1 Viability assays

Cells were seeded in 96‐well transparent round‐bottomed plates (4000 cells per well) and grown for 24 h. Cells were then treated with culture medium either supplemented with SO1861 or not. The safely tolerated concentration of SO1861 on cells after 72 h was determined by 3‐(4,5‐dimethylthiazol‐2‐yl)‐2,5‐diphenyltetrazolium bromide (MTT). Thereafter, viability of ^His^Dianthin (final concentration 1 pm to 1 μm) and ^His^Dianthin‐EGF (final concentration 0.1 pm to 1 μm) either supplemented with SO1861 or not after 48 h was determined by MTT. Controls were treated only with culture medium or 0.5 μg·mL^−1^ SO1861. Experiments with SO1861 variation were conducted at the determined IC_50_ concentration of ^His^Dianthin‐EGF obtained in the presence of 0.5 μg·mL^−1^ SO1861 for each cell line (Table 1).

**Table 1:** Specificity of ^His^Dianthin‐EGF in the absence and presence of SO1861. The table shows IC_50_ values calculated from corresponding cytotoxicity assays conducted as described in the methods section, and derived targeting indices and SO1861 enhancement factors. TI, targeting index = IC_50_ (toxin)/IC_50_ (targeted toxin); EF, enhancement factor = IC_50_ (−SO1861)/IC_50_ (+SO1861); D, ^His^Dianthin; DE, ^His^Dianthin‐EGF

Cell lines	IC_50_	IC_50_	TI	IC_50_	IC_50_	TI	EF	EF
	D (nm)	DE (nm)	D/DE	D + SO1861 (nm)	DE + SO1861 (nm)	D/DE + SO1861	D	DE
BxPC‐3	10 900	100	109	390	0.17	2294	28	588
MIA PaCa‐2	100	30	3.3	40	5.3	7.5	2.5	5.7
NIH‐3T3	22	73	0.3	186	110	1.7	0.12	0.7

#### 2.4.2 Real‐time monitoring

Real‐time monitoring of the cell growth after treatment with SO1861 in combination with ^His^Dianthin‐EGF was carried out using the xCELLigence real‐time cell analyzer (Roche, Basel, Switzerland). With this device, cell proliferation can be recorded continuously during the experiment by measuring the impedance of the cells that are attached on the surface of cell culture plates with interdigitated gold microelectrodes (e‐plates).

NIH‐3T3, MIA PaCa‐2, and BxPC‐3 cells were trypsinized by using trypsin/EDTA (0.25%). Meanwhile, the blank measurement (50 μL DMEM, or RPMI‐1640 for BxPC‐3) was performed on 96‐well plates (16‐well for BxPC‐3) in the xCELLigence device. Thereafter, 4000 cells/100 μL were seeded per well. After 24 h when the impedance‐based cell index reached between 0.5 and 1, ^His^Dianthin‐EGF with and without SO1861 was added in triplicate (in duplicate for BxPC‐3) in different concentrations (100 pm to 1 μm for single treatment and 10 pm to 100 nm in case of combination treatment with SO1861). After 120 h of real‐time measurement, cytotoxicity was evaluated using the xcelligence software (RTCA 2.0; ACEA Biosciences Inc., San Diego, CA, USA).

All the *in vitro* experiments, unless otherwise mentioned in the legend, were performed in quadruplicate in three independent experiments, and the values are reported as their mean.

### 2.5 *In vivo* studies

All animal experiments were approved by the local authority according to national animal ethics regulations (approval number G 0260/10 from LAGeSo, Berlin, Germany). The recommendations of the animal welfare officer of the Charité, Berlin, were strictly adhered to during the entire duration of the experiment.

#### 2.5.1 Acute toxicity study

Acute toxicity studies were performed on groups of 7‐month‐old male BALB/c mice weighing 30–35 g. The study comprised four groups each containing three mice. Doses of 40, 4, and 0.4 μg ^His^Dianthin‐EGF in 100 μL PBS were administered once per mouse intraperitoneally in three groups, and the fourth group was the nontreated control group. Thereafter, the mice were observed every 1 h on the first day and then twice every 24 h for their body weight and behavioral changes for 1 week. Animals were then sacrificed and organs were collected and prepared for histopathological analysis.

### 2.6 Xenograft studies

#### 2.6.1 Development of a solid tumor xenograft model

To develop a tumor growth rate curve, 1.25 × 10^6^ BxPC‐3 cells/100 μL in PBS/mouse were injected subcutaneously into the right flank of six 7‐month‐old male CD‐1 nu/nu mice (28–35 g; Charles Rivers, Sulzfeld, Germany). Animals were observed daily for their body weight shifts and tumor development. After 13 days, a small palpable tumor was observed, although not measurable using vernier caliper. Two days later, tumor size was measured by caliper and the process was followed, twice weekly for 4 weeks. Animals were then sacrificed and tumor growth rate curve was established. The EGFR expression level was determined histologically for the grown tumors.

#### 2.6.2 Efficacy of ^His^Dianthin‐EGF alone or in conjunction with SO1861 as compared to placebo

The *in vivo* efficacy experiments were carried out in 7‐month‐old CD‐1 nu/nu mice, comprising five animals per group. The mice were housed in individually ventilated cages under a constant day and night cycle (12 h each) and had free access to animal feed (Ssniff, Soest, Germany) and water. All animals were monitored daily for health and well‐being during the entire experiment. In order to slightly elevate tumor growth, the tumor was inducted by a subcutaneous injection of 1.3 × 10^6^ BxPC‐3 cells at the right flank of each animal with sufficient distance to the vertebral column. The cells were diluted in 100 μL PBS and injected with a 29‐gauge needle.

Based on our previous toxicological experiments (Bachran *et al*., 2010a), the dosage of SO1861 was fixed at 30 μg per treatment diluted in 100 μL Dulbecco's PBS without Ca^2+^ and Mg^2+^ (PAA, Linz, Austria), which was applied subcutaneously into the neck. A total of 60 min later, 0.35 μg ^His^Dianthin‐EGF (subcutaneously in the back, in the vicinity of the tumor) again in 100 μL PBS was injected. The control group was treated with PBS alone administered in the neck and in the back. In some individuals of all groups, the tumor developed slightly shifted from the right flank to the back so that injection in the vicinity of the tumor also includes a position at the back near the tumor. The therapy was started after randomly assigning the animals to three different groups of five animals each on day 7 when a palpable tumor of approximately 2 mm was formed at the site of injection of tumor cells in all the animals. In total, there were six therapy cycles per mouse carried out on days 7, 10, 13, 16, 19, and 22 after tumor cell injection. The growth of the tumor was monitored and the final tumor volume was determined with the help of digital vernier caliper.

### 2.7 Hematological analysis

Approximately 1.2 mL of blood was collected by cardiac puncture on the 25th day of the experiment in isoflurane‐anesthetized mice. Blood was collected in an S‐Monovette^®^ 1.2‐mL K3 EDTA sterile tubes and was sent to Labor 28 GmbH, Berlin, Germany, for complete blood count analysis, including red and white blood cell counts, platelets, hemoglobin, hematocrit, mean corpuscular volume, mean corpuscular hemoglobin, and mean corpuscular hemoglobin concentration.

### 2.8 Histopathological analysis

The animals were sacrificed by cervical dislocation. The tumors were collected along with the adjoining skin. In case that no tumor was macroscopically seen, the skin from the site of tumor cell injection was prepared. In addition, the spleen, liver, lung, heart, intestine, and kidneys were also collected. After formol fixation, the organs and the tumor samples were embedded in paraffin for histological examination. The samples were cut into 4‐μm slices and were stained with hematoxylin–eosin (HE) and immunohistologically for the determination of the EGFR expression level (EGFR pharmDx™ Kit; DAKO, Hamburg, Germany). Ki‐67 proliferation index (monoclonal antibody Ki‐67P; Dianova, Hamburg, Germany) was also determined for the tumor samples and detected with secondary reagents (K5005; DAKO). The tissues were examined for any histopathological alterations, which might be due to toxin‐induced injury using the criteria as published elsewhere (von Mallinckrodt *et al*., 2014).

### 2.9 Statistical analyses

All the statistical analyses were performed with the help of graphpad prism 6.0. Student's *t*‐test was utilized for the examination of significant difference in cytotoxicity assays. Paired and unpaired *t*‐tests using graphpad software were used to evaluate significance level in body weight loss and dose–response curve studies in xenografts, respectively. The concentration that inhibits cell survival to half maximal extent (IC_50_) was calculated by the method of least squares using a sigmoidal four‐parameter logistic regression.

## 3 Results

### 3.1 Purification and determination of enzymatic activity of ^His^Dianthin‐EGF

**Figure 1:**
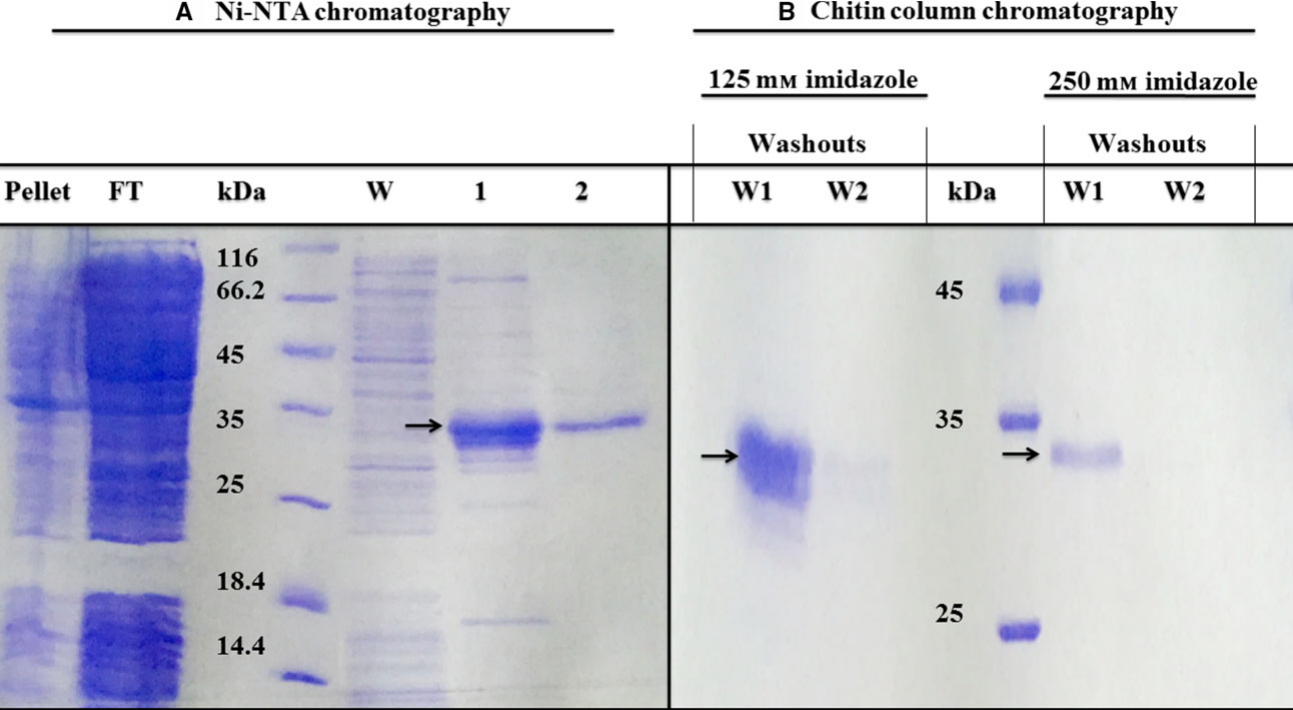
SDS/PAGE (12%) of the pellet obtained after ultrasonication and purified fractions of proteins obtained after (A) Ni‐NTA chromatography and (B) chitin column affinity chromatography. FT, flow‐through; W, washout; lane ‘1’: 125 mm imidazole eluate; lane ‘2’: 250 mm imidazole eluate. Arrows point to ^His^Dianthin‐EGF.

In a first step, the recombinantly expressed ^His^Dianthin‐EGF was purified by Ni‐NTA chromatography. Obtained fractions were analyzed by SDS/PAGE (Fig. 1A). No substantial amounts of ^His^Dianthin‐EGF were found in the pellet obtained after ultrasonication, the flow‐through of the column, and the washout. At 36 kDa, a clear band of ^His^Dianthin‐EGF was detected in the 125 mm imidazole eluate, but slight contaminations of bacterial proteins were also observed. As the 250 mm imidazole eluate also contained some ^His^Dianthin‐EGF, both fractions were dialyzed and then applied to a chitin column chromatography to remove contaminating proteins. SDS/PAGE revealed highly purified ^His^Dianthin‐EGF with no contaminants visible in the Coomassie stain (Fig. 1B). The identity was verified by western blotting (Fig. S1) and former preparations also by matrix‐assisted laser desorption/ionization‐time of flight (Gilabert‐Oriol *et al*., 2013c). The total yield for ^His^Dianthin‐EGF was around 8 mg from 2 L of bacterial suspension. In an enzymatic activity assay for ribosome‐inactivating proteins, recombinant ^His^Dianthin‐EGF released 42 pmol adenine·pmol toxin^−1^·h^−1^ compared to 67 pmol·pmol^−1^·h^−1^ for ligand‐free ^His^Dianthin, indicating 37% loss in catalytic activity due to fusion to EGF.

### 3.2 Cytotoxicity of ^His^Dianthin and ^His^Dianthin‐EGF augmented by SO1861

**Figure 2:**
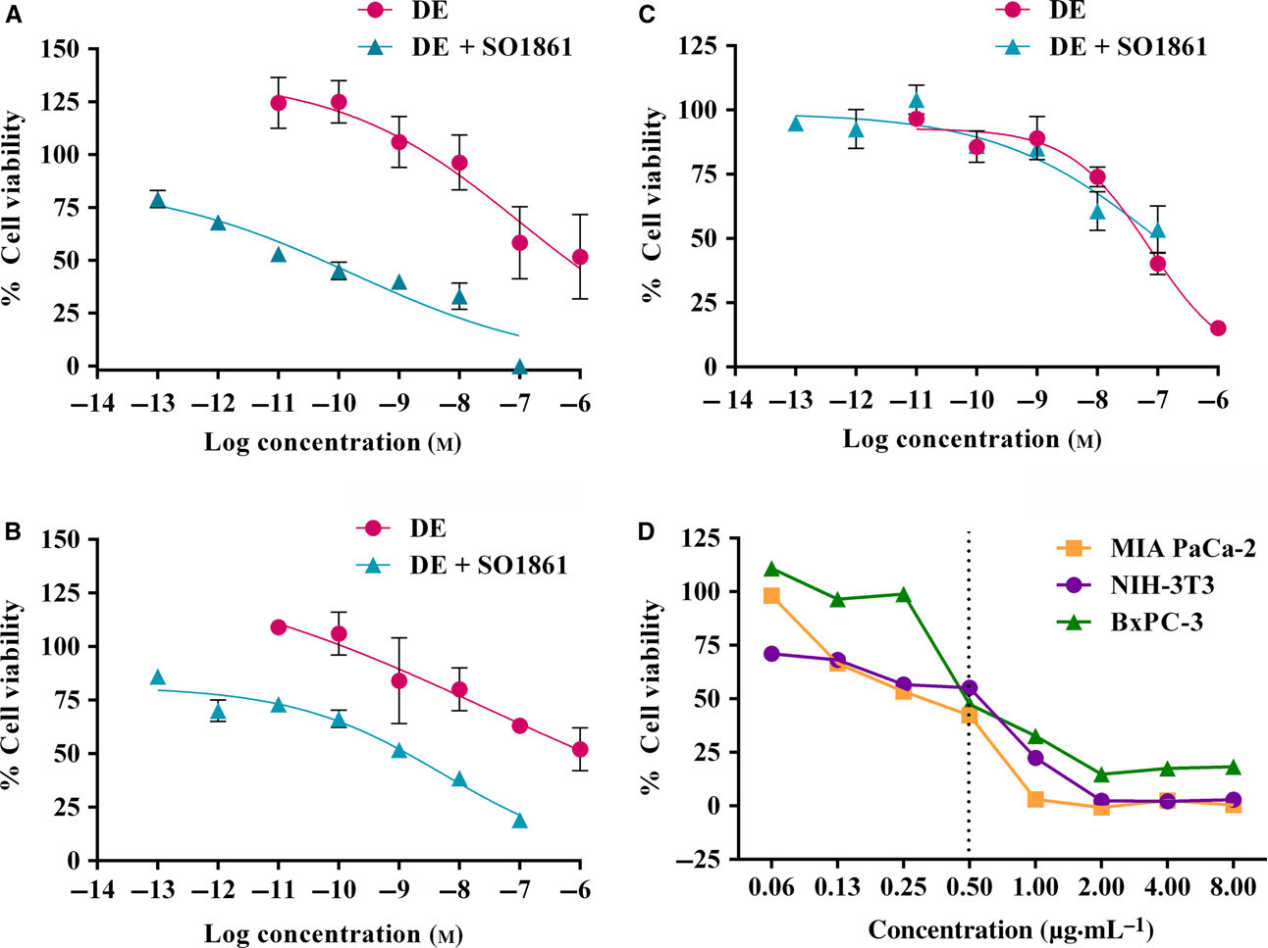
SO1861‐mediated augmentation of ^His^Dianthin‐EGF (DE). (A, D) BxPC‐3, (B, D) MIA PaCa‐2, and (C, D) NIH‐3T3 cells were seeded in 96‐well plates and grown for 24 h. Cells were then treated with (A–C) varying concentrations of ^His^Dianthin‐EGF in DMEM (MIA PaCa‐2, NIH‐3T3) or RPMI 1640 (BxPC‐3) in the presence and absence of SO1861 (0.5 μg·mL^−1^), or (D) at a fixed concentration of ^His^Dianthin‐EGF corresponding to the IC_50_ shown in Table 1 and varying concentrations of SO1861. Cells were further incubated for 48 h. Viability was determined by an MTT assay. Each value represents the mean of three (two for panel (D)) independent experiments performed in quadruplicate. Single outliers defined as outside ± 3‐fold standard deviation were omitted in (D). A statistical significant effect between single and combination treatments was observed in MIA PaCa‐2 (Student's *t*‐test; *P* < 0.05) and BxPC‐3 (Student's *t*‐test; *P* < 0.01) cell lines. The vertical line in (D) indicates the SO1861 concentration used in (A–C) to determine the IC_50_ of ^His^Dianthin‐EGF and is thus the expected IC_50_ for the variation in SO1861.

To first characterize efficacy and selectivity of ^His^Dianthin‐EGF on pancreatic cell lines and the enhancer effect of SO1861, cytotoxicity of ^His^Dianthin‐EGF in the absence and presence of SO1861 was determined on the EGFR‐overexpressing pancreatic carcinoma cell lines BxPC‐3 and MIA PaCa‐2 as well as on off‐target NIH‐3T3 cells. The expression of EGFR in BxPC‐3 cells was 1.7‐fold higher than in MIA PaCa‐2 cells (Fig. S5). The IC_50_ of ^His^Dianthin‐EGF in the absence of SO1861 was found to be 1.0 × 10^−7^ m for BxPC‐3 (Fig. 2A) and 3.0 × 10^−8^ m for MIA PaCa‐2 (Fig. 2B) cells after 48 h. Similarly, NIH‐3T3 cells revealed an IC_50_ of 7.3 × 10^−8^ m (Fig. 2C), indicating no relevant difference between target and off‐target cells. Nevertheless, the targeting index shows that ^His^Dianthin‐EGF is specific for EGFR‐expressing cells and the toxic effect on NIH‐3T3 cells can be attributed to its intrinsic sensitivity to ^His^Dianthin (see end of this section). For the combination treatment with SO1861, a safe concentration of the enhancer alone was determined first by a concentration variation in the range from 0.13 to 32 μg·mL^−1^ by end‐point assays (Fig. S2) and in addition by real‐time assays for BxPC‐3 cells in the range from 0.2 to 10 μg·mL^−1^ (Fig. S6). A safe concentration was defined as a concentration that does not show any toxic effect and for that the twofold of this concentration shows more than 90% cell survival. In the presence of SO1861 (0.5 μg·mL^−1^), a clear enhancement of the toxicity within 48 h was observed on target cells as compared to the off‐target cell line (IC_50_ of 1.7 × 10^−10^ m for BxPC‐3, 5.3 × 10^−9^ m for MIA PaCa‐2 and 1.1 × 10^−7^ m for NIH‐3T3 cells, Fig. 2A–C). This clearly indicated a cytotoxic synergism between ^His^Dianthin‐EGF and SO1861 on pancreatic tumor cell lines. Notably, the synergistic effect is target cell specific, suggesting that ^His^Dianthin‐EGF is also internalized successfully in the absence of SO1861 but is ineffective due to insufficient endosomal escape, which is then triggered by SO1861 in the combined treatment. The corresponding enhancement factors are represented by the ratios of the IC_50_ values in the absence and presence of SO1861. We calculated 0.7 for off‐target NIH‐3T3 cells and 588 and 5.7 for BxPC‐3 and MIA PaCa‐2 cells, respectively (Table 1). A ratio close to 1 as observed for NIH‐3T3 cells stands for no difference; that is, no substantial amount of targeted toxins has reached the endosomes, indicating receptor‐dependent specificity. It is also important to mention that SO1861 itself does not have any selectivity to EGFR‐expressing cells. When investigating the effect of an SO1861 concentration variation at the fixed IC_50_ concentration of ^His^Dianthin‐EGF (Table 1) for all cell lines, no significant difference can be observed for all cell lines independent of target receptor expression (Fig. 2D). Thus, the observation that SO1861 specifically enhances the effect of ^His^Dianthin‐EGF in target cells is solely attributed to the target cell specificity of ^His^Dianthin‐EGF. SO1861 only ensures that the toxin takes full effect (by mediating endosomal escape). Although high concentrations of SO1861 show better effects (Fig. 2D), these concentrations cannot be used for treatment as part of this effect is caused by the intrinsic toxicity of SO1861, which is not target cell specific (Fig. S2). Therefore, SO1861 is always used at a nontoxic concentration.

The specificity of ^His^Dianthin‐EGF for target cells was also shown by comparison with the ligand‐free toxin ^His^Dianthin alone. The IC_50_ values that have been calculated from cytotoxicity curves were used to calculate the targeting index (IC_50_ of ^His^Dianthin divided by IC_50_ of ^His^Dianthin‐EGF) (Table 1). The targeting index is much more better for BxPC‐3 than for MIA PaCa‐2 cells corresponding to the EGFR expression in these cell lines (Fig. S5). The index less than 1 in off‐target NIH‐3T3 cells might be traced back to the bigger size of the fusion protein, which most likely results in reduced unspecific uptake. The completely different IC_50_ values of ^His^Dianthin for the examined cell lines reflect the different unspecific cytosolic uptake of ^His^Dianthin and varying intrinsic sensitivity for this toxin. The enhancement factors of SO1861 for ^His^Dianthin are greater than 1, indicating unspecific enhancement, but less than for ^His^Dianthin‐EGF (Table 1), indicating in total a gain in specificity as already shown for another ribosome‐inactivating protein, ^His^Saporin‐EGF compared to ^His^Saporin (Bachran *et al*., 2010b).

### 3.3 Real‐time monitoring of ^His^Dianthin‐EGF cytotoxicity

**Figure 3:**
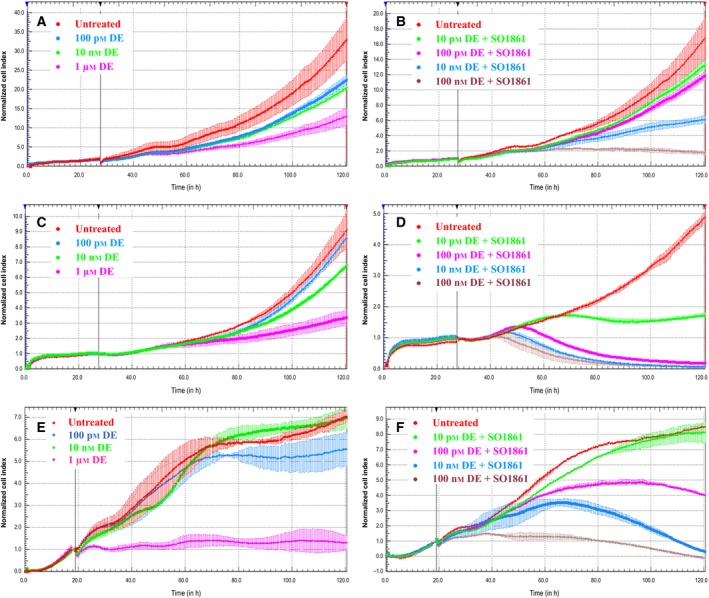
Real‐time cell analysis showing the dose‐dependent increase in cytotoxicity in monotherapy with ^His^Dianthin‐EGF (DE) (A, C, E) and in combination therapy together with SO1861 (B, D, F) in EGFR‐overexpressing MIA PaCa‐2 cells (C, D) and BxPC‐3 cells (E, F) as compared to NIH‐3T3 cells (A, B), which is an off‐target cell line. The *y*‐axis shows the impedance‐based cell index that was normalized to 1.0 after 28 h in case of NIH‐3T3 and MIA PaCa‐2 cells and, due to faster cell proliferation, after 19 h in case of BxPC‐3 cells just before treatment started. The cell index can be assumed to be proportional to the number of living cells.

The kinetics of ^His^Dianthin‐EGF cytotoxicity was evaluated in real time in all three cell lines (Fig. 3). A gradual increase in the impedance signal (i.e., cell growth) compared to the normalized cell index was observed when cells were treated with the targeted toxin in the absence of SO1861 at 100 pm, 10 nm, and 1 μm except for BxPC‐3 cells when treated with 1 μm where a cytostatic effect was observed (Fig. 3A,C,E). Cell growth was reduced beginning 30 h after toxin incubation (3 h for BxPC‐3 cells at 1 μm). The reduction was dose dependent and continued until the end of the experiment (92 h after induction) but there was still cell proliferation at any time except for BxPC‐3 cells at 1 μm. In contrast, a dose‐dependent decrease in the normalized cell index (i.e., cell death) was observed with the combined application of SO1861 and ^His^Dianthin‐EGF (Fig. 3B,D,F), finally leading to complete cell death for MIA PaCa‐2 cells 72–92 h after beginning of the treatment for concentrations of 100 nm, 10 nm, and 100 pm of the targeted toxin, while off‐target NIH‐3T3 cells only show a decreasing signal at 100 nm (Fig. 3B). BxPC‐3 cells do not reach complete cell death in the observed time period; however, the signal is still decreasing at the end of the experiment and it must be taken into consideration that untreated BxPC‐3 cells proliferate much faster than MIA PaCa‐2 cells. The lowest concentration of 10 pm ^His^Dianthin‐EGF resulted only in a cytostatic effect 20 h after start of the incubation for MIA PaCa‐2 cells and in no effect for BxPC‐3 cells. The kinetics of the first observable effect was faster in the combination therapy (14–24 h after treatment start for concentrations down to 100 pm) compared to the monotherapy (30 h, except for BxPC‐3 cells at 1 μm). The enhancer effect of SO1861 is target cell specific as cell growth of NIH‐3T3 cells was not substantially more reduced than with the monotherapy (Fig. 3A,B) and cell proliferation was observed for all concentrations except for the highest concentration of 100 nm that resulted in cytostatic behavior.

### 3.4 Evaluation of effect levels by acute toxicity study

**Figure 4:**
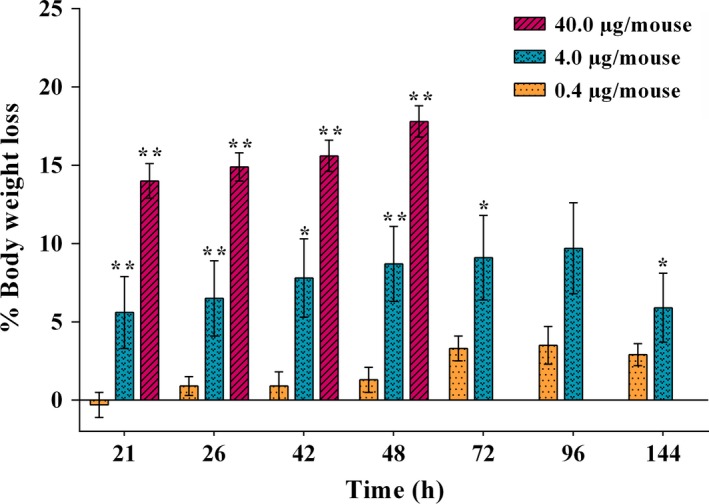
Body weight loss observed after single application of different doses of ^His^Dianthin‐EGF. The values refer to the body weight just before treatment. Significance in body weight changes is indicated by * (*P* < 0.05) and ** (*P* < 0.01).

No acute toxic effects were observed in former studies for mice treated only with a comparable enhancer (Saponinum album) up to 100 μg (Heisler *et al*., 2005). For ^His^Dianthin‐EGF alone, 0.4 μg per mouse was found to be nontoxic. The body weight remained constant throughout the week after single dose administration, while for 4.0 μg per mouse, a statistical significant but mild decrease in body weight was observed with no adverse effects as defined in Table S1. However, 40 μg dose caused moribundity accompanied with white ocular discharge in 100% population (Fig. S3). Furthermore, a decrease in more than 10% body weight within 48 h was also observed (Fig. 4). The animals were sacrificed (after 48 h for 40 μg dose and after 1 week for 0.4 and 4.0 μg doses) and organs were preserved for further toxicological analyses by immunohistochemistry.

### 3.5 Efficacy of ^His^Dianthin‐EGF alone and in combination with SO1861 as compared to placebo

**Figure 5:**
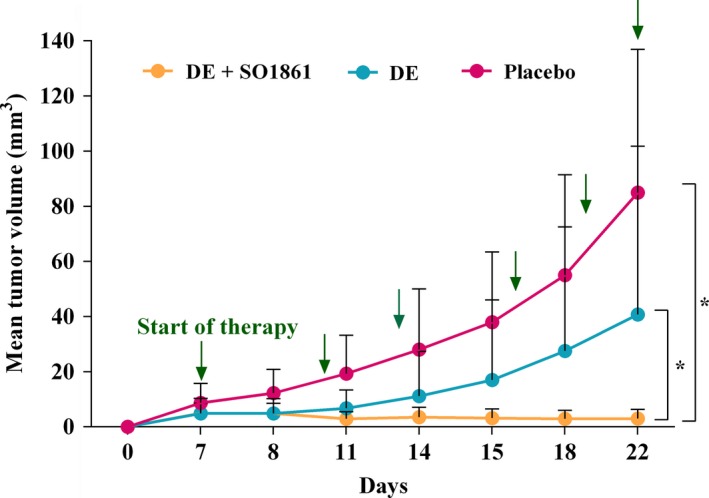
Xenograft tumor volume evaluated by the use of a digital vernier caliper for placebo (PBS only), single (^His^Dianthin‐EGF; 0.35 μg per treatment), and combination (^His^Dianthin‐EGF; 0.35 μg per treatment + SO1861; 30 μg per treatment) therapy. Arrows point to the days of treatment. A statistical significant decrease in tumor volume was observed in the combination treatment for the curves as a whole (*P* < 0.05 versus single therapy and versus placebo).

The therapeutic effect of the combination therapy in tumor‐bearing nude mice was determined for a period of 25 days comprising six treatment cycles. The total amount was therefore six times 0.35 μg ^His^Dianthin‐EGF; that is, 2.1 μg. Taking into consideration that 4 μg in a single dose did not result in adverse effects, we assumed the applied dose to be safe. There was a 96.5% average reduction in the tumor volume for the group treated with the combination of ^His^Dianthin‐EGF and SO1861 compared to the placebo (tumor volume 84.5 ± 51.9 mm^3^ for placebo and 3.0 ± 3.3 mm^3^ for combination) and four of five mice showed complete regression (Fig. 5). The monotherapy with ^His^Dianthin‐EGF also caused a decrease in tumor volume (40.8 ± 61.3 mm^3^), which was a 51.7% average reduction when compared to placebo; however, treatment together with SO1861 resulted in a more than 13‐fold better efficacy. As a nonsevere side effect, SO1861‐induced skin hardening was observed at the back of the neck after two therapy cycles. The endosomal escape enhancer alone has no effect on tumor growth as shown in previous studies (Bachran *et al*., 2009).

**Figure 6:**
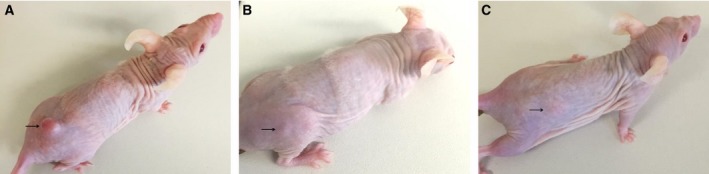
Images depicting tumor volumes and lesion‐free skin after six therapy cycles of (A) placebo, (B) ^His^Dianthin‐EGF, and (C) ^His^Dianthin‐EGF and SO1861. Arrows point to the injection site of tumor cells. The pictures show one representative animal of each group comprising five mice.

No complete regressions were observed in the monotherapy, two mice had continuous tumor growth, and three had retarded tumor growth. The experiment was brought to an end after six therapy cycles and mice curatively treated with the combination therapy did not show any skin lesions neither at the injection nor at the tumor site (Fig. 6).

### 3.6 Histological and immunohistochemical analyses

**Figure 7:**
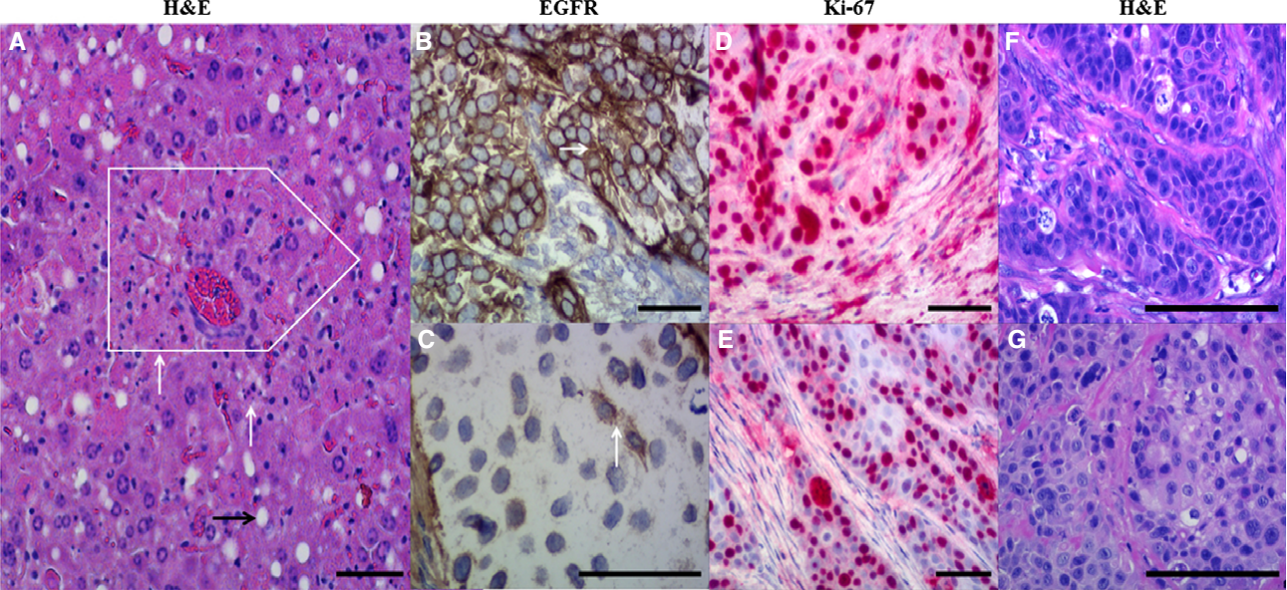
Histological and immunohistochemical evaluation of (A) liver and (B–G) tumor tissue samples. Hematoxylin and eosin (H&E) staining (A) showed single‐cell necrosis (white arrows) and necrosis of small groups of hepatocytes (white box); that is, moderate damage of liver tissues isolated from mice administered with the highest single dose of ^His^Dianthin‐EGF (40 μg). (B) High EGFR expression level (arrow) was observed in untreated tumors, whereas (C) a weak expression of EGFR (arrow) was observed in the treatment group depicting curative effect of therapy as compared to untreated. High Ki67 proliferation index (D) was observed in all tumors except the one obtained after combination therapy [50%; (E)]. The right row of photographs (F, G) represents H&E‐stained samples corresponding to areas of the immunohistologically EGFR‐ and Ki‐67‐stained samples. Part (F) shows the tumor of an untreated mouse, whereas part (G) depicts the tumor of the sole mouse that retained a tumor after treatment with ^His^Dianthin‐EGF + SO1861. The immunohistochemical samples are counterstained by hematoxylin. All scale bars represent a length of 100 μm.

To evaluate acute toxicity, histopathological analyses of isolated organs by HE stains were performed. Most of the organs did not reveal any alterations and were labeled as regular; especially, no alteration was seen in stomach, heart, and intestine of any mice. However, mild to moderate alterations like single‐cell necrosis and necrosis of groups of hepatocytes were observed in liver tissue of mice, in which the highest dose (40 μg per mouse) was administered (Fig. 7A). Nevertheless, no severe damage was observed.

Tumors isolated from the efficacy studies accomplished in CD1 nu/nu xenografts were immunohistochemically analyzed for the expression of EGFR and the Ki67 proliferation marker. All isolated tumors from the placebo group expressed high levels of EGFR (Fig. 7B); however, only a weak EGFR staining was examined in mice treated with the monotherapy (Fig. 7C), indicating that the monotherapy is only suitable to kill tumor cells with high EGFR expression, while tumor cells with lower EGFR expression escape from treatment and selectively proliferate. We also tested EGFR expression in cell culture experiments by western blotting, but were unable to detect significant differences after 24, 48, and 72 h. Due to the toxicity of the treatment, longer observation periods were not possible in cell culture. The different EGFR expression in tumors might have been developed in an evolutionary selection process during the long period of 4‐week treatment.

A high Ki67 proliferation index (> 60%; Fig. 7D) was observed in all isolated tumors of the placebo and monotherapy group. This let us assume that the reduced tumor size in the monotherapy group can be attributed only to the killing of high EGFR‐expressing tumor cells but not to a lower proliferation rate of the tumor cells with lower EGFR amount. In the combination therapy after six treatment cycles, only one of five mice still had a tumor that could be investigated for the growth capacity. This tumor only had a 50% Ki67 proliferation index (Fig. 7E).

### 3.7 Complete blood count analyses

**Figure 8:**
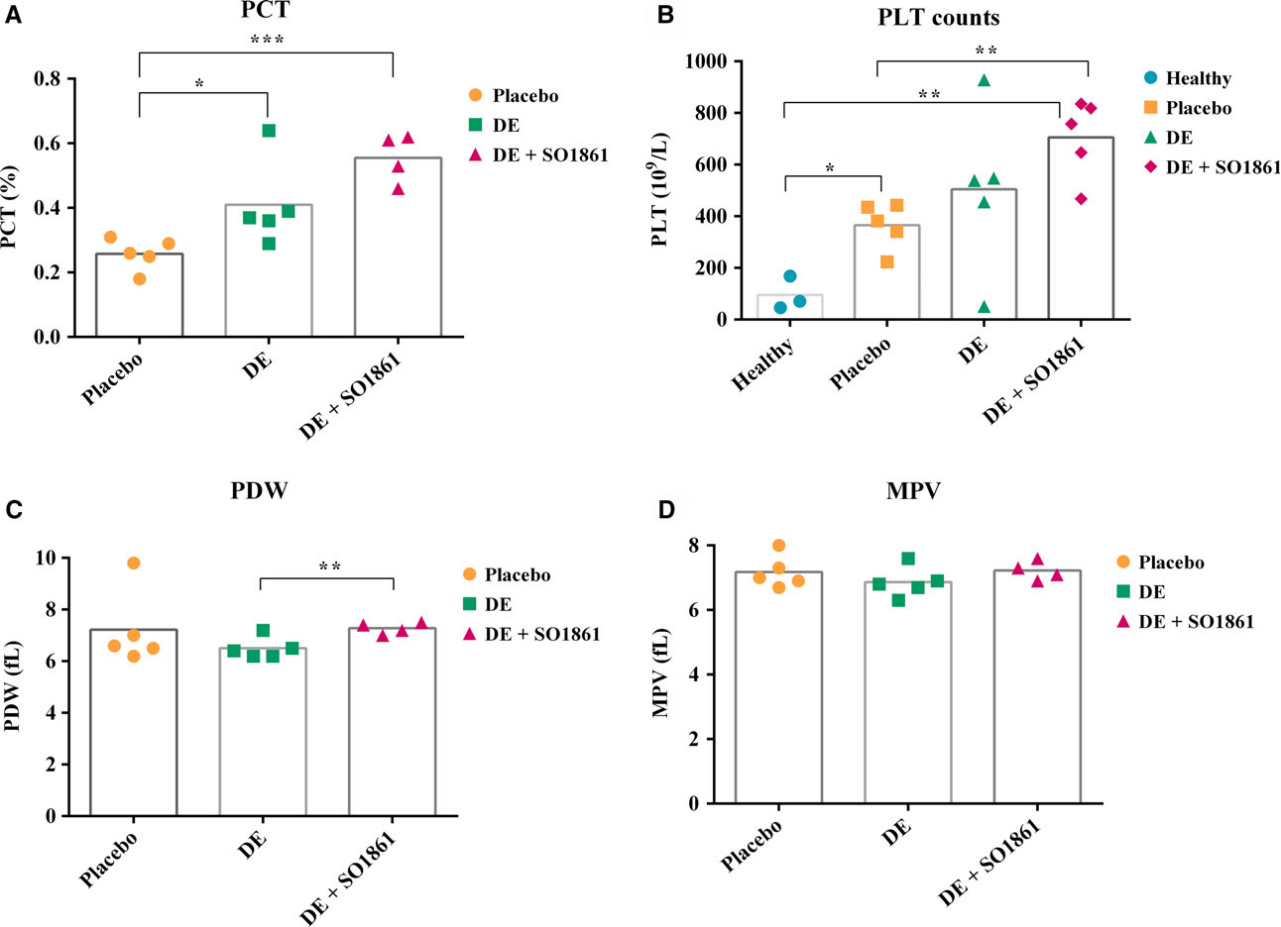
Platelet‐derived parameters in different groups (healthy, placebo, ^His^Dianthin‐EGF, and ^His^Dianthin‐EGF + SO1861 treated). Significance is indicated as * (*P* < 0.05), ** (*P* < 0.01), and *** (*P* < 0.001). PLT counts, platelet counts; PCT, plateletcrit; PDW, platelet distribution width; MPV, mean platelet volume.

No significant difference was seen in the non‐platelet‐derived parameters of blood as displayed in Table 2. In contrast, the platelet‐derived parameters showed statistically significant alterations as depicted in Fig. 8.

**Table 2:** Descriptive statistics of non‐platelet‐derived hematological parameters. None of the differences are statistically significant. DE, ^His^Dianthin‐EGF; RBC, red blood cell count; WBC, white blood cell count; HGB, hemoglobin; RDW‐SD, red blood cell distribution width standard deviation; RDW‐CV red blood cell distribution width coefficient of variation; HCT, hematocrit; MCV, mean corpuscular volume; MCH, mean corpuscular hemoglobin; MCHC, mean corpuscular hemoglobin concentration

Parameters	Healthy	Placebo	DE	DE + SO1861
WBC	2.3 ± 0.5	3.2 ± 0.3	2.4 ± 0.8	3.0 ± 0.8
RBC	7.7 ± 1.0	7.7 ± 0.4	7.6 ± 0.6	7.1 ± 1.0
HGB	11.8 ± 1.4	11.6 ± 0.6	11.3 ± 0.8	10.9 ± 1.0
RDW‐SD	26.5 ± 2.7	26.8 ± 1.8	26.8 ± 2.4	32.2 ± 6.0
RDW‐CV	16.3 ± 2.0	16.3 ± 0.6	17.0 ± 0.2	18.0 ± 0.9
HCT	38.5 ± 1.0	39.0 ± 0.9	37.3 ± 1.2	37.8 ± 1.4
MCV	50.0 ± 0.9	50.7 ± 1.9	48.9 ± 2.3	53.5 ± 6.5
MCH	15.4 ± 0.3	15.1 ± 0.6	14.8 ± 0.3	15.3 ± 1.1
MCHC	30.8 ± 1.0	29.8 ± 0.9	30.3 ± 1.2	28.7 ± 1.4

A significant increase in platelet count was observed in case of the combination treatment (*P* < 0.01 versus healthy as well as placebo). Surprisingly, there was also a significant increase in platelet count observed for placebo when compared to healthy (*P* < 0.05), indicating that the puncture wound has some influence on the platelets. An increase in plateletcrit was also significant compared to placebo (*P* < 0.05 versus ^His^Dianthin‐EGF; *P* < 0.0001 versus ^His^Dianthin‐EGF + SO1861). A slight but significant difference in platelet distribution width was observed for monotherapy compared to the combination (*P* < 0.05), while the mean platelet volume showed no significant difference.

## 4 Discussion

Recombinant expression of targeted ribosome‐inactivating proteins in chemically competent *E. coli* Rosetta (DE3) pLysS cells had already been described in previous studies (Gilabert‐Oriol *et al*., 2013c; Heisler *et al*., 2005). The purity of proteins plays an important role and poly‐histidine‐tagged recombinant proteins that are isolated by immobilized metal affinity chromatography are often contaminated with significant amounts of endogenous *E. coli* metal‐binding proteins. Several techniques were developed to solve this problem including the variation in the poly‐histidine tags and the applied detergent (Grisshammer and Tucker, 1997), use of particular matrices such as nitrilotriacetic acid (Hochuli *et al*., 1987) and Co^2+^‐carboxymethylaspartate (Chaga *et al*., 1999), or by the use of particular *E. coli* strains such as NiCo21(DE3) and NiCo22(DE3) that allow removal of contaminating *E. coli* proteins by the use of a chitin column (Robichon *et al*., 2011). We aimed to minimize this contamination by using the NiCo21(DE3) *E. coli* strain considering it to be a superior alternative to BL21(DE3) (Robichon *et al*., 2011). Although this led to 2.7‐fold decrease in enzymatic activity as compared to an earlier study described (von Mallinckrodt *et al*., 2014), the targeted toxin was now highly pure and retained sufficient enzymatic activity. The decrease in activity might be attributed to partly improper folding of ^His^Dianthin‐EGF expressed in NiCo21(DE3) compared to BL21(DE3). Nevertheless, to avoid the proinflammatory effects observed by bacterial contaminants (Pepys *et al*., 2005), more emphasis on purity was given.

EGFR‐targeting toxins in synergism to the endosomal escape mechanism of SO1861 might have an important regulatory role in the control of tumor growth in xenografts bearing human adenocarcinoma pancreatic tumors. A strong evidence for this hypothesis is based on *in vitro* (Bachran *et al*., 2011; Gilabert‐Oriol *et al*., 2015; Weng *et al*., 2012) as well as *in vivo* studies that were previously performed in BALB/c and nude mice demonstrating very strong synergistic effects of the combination therapy for mammary and colon carcinoma (Bachran *et al*., 2009; Gilabert‐Oriol *et al*., 2013c; von Mallinckrodt *et al*., 2014). The aim of our study was to investigate whether the combinatory treatment with SO1861 is also suitable to treat pancreatic adenocarcinoma, which is characterized by aggressive growth and a very low overall five‐year survival rate of less than 4% (Kleeff *et al*., 2006). When we compared saporin‐ and dianthin‐based targeted toxins, we observed that dianthin‐based products were much more stable (Gilabert‐Oriol *et al*., 2015) and produced better protein yield (Gilabert‐Oriol *et al*., 2013c) as compared to saporin‐based targeted toxins (Gilabert‐Oriol *et al*., 2013b). Moreover, there are hints that dianthin is less immunogenic than saporin (Strocchi *et al*., 1992). Therefore, we will conduct future investigations with the aim of clinical studies with dianthin‐based products. Moreover, side effects of this therapy were not sufficiently perused to date and we therefore investigated hematological parameters after mono‐ and combination therapy of ^His^Dianthin‐EGF in particular with regard to the known hemolytic properties of some glycosylated triterpenoids in general (Gilabert‐Oriol *et al*., 2013a,c).

In the present study, EGF serves as a ligand to target EGFR molecules. Cytotoxicity was therefore evaluated in EGFR‐overexpressing human pancreatic carcinoma cells (BxPC‐3 and MIA PaCa‐2). Higher expression of EGFR had been found in BxPC‐3 cells in comparison with MIA PaCa‐2 as demonstrated by western blot (Fig. S5) (Ali *et al*., 2005) and also by flow cytometry (Ioannou *et al*., 2011). *In vitro*, we found no significant difference between the cytotoxic effect on target and off‐target cells when cells were treated in the absence of SO1861 (Fig. 2). Addition of SO1861 solely enhanced the cytotoxic effect on target cells, indicating that ^His^Dianthin‐EGF enters off‐target cells via unspecific routes omitting late endosomes (explaining the absent enhancer effect of SO1861), while late endosomes are expectedly reached in target cells, but no increased cytotoxic effect compared to off‐target cells is observed due to missing endosomal escape, which only occurs in the presence of SO1861 (Fig. 2A,B). ^His^Dianthin‐EGF‐induced growth inhibition in the presence of SO1861 varied between different target cell lines. As the expression level of EGFR is high in BxPC‐3 in comparison with MIA PaCa‐2 (Fig. S5), it can be expected that the augmentation of the endosomal escape by SO1861 results in a stronger efficacy increase in BxPC‐3 cells. Indeed, the difference in the cytotoxic effect between single and combination therapy proved to be more eminent in BxPC‐3 cells, as portrayed by an IC_50_ of 100 nm in monotherapy and 0.17 nm in combination therapy within 48 h. Studies on saporin‐based targeted toxins showed that the IC_50_ is strongly dependent on target receptor expression and can range from 2.5 nm to 206 nm in the absence of endosomal escape enhancers (Bachran *et al*., 2010b). For ^His^Dianthin‐EGF, the IC_50_ was only 0.45 nm on transfected cells that highly overexpress EGFR (Weng *et al*., 2012) and greater than 10 nm for ^His^Dianthin chemically conjugated to panitumumab, trastuzumab, or cetuximab (Gilabert‐Oriol *et al*., 2015). The effect by endosomal escape enhancers depends on the cell line, the targeting moiety, target receptor expression, and the structure of the enhancer. Enhancement factors of more than one million were observed (Weng *et al*., 2012). The IC_50_ for the above‐mentioned antibody–^His^Dianthin conjugates in the presence of SO1861 was 1.5, 23, and 5.3 pm, respectively (Gilabert‐Oriol *et al*., 2015). Real‐time cytotoxicity studies also revealed the combination treatment to be more superior compared to monotherapy with only targeted toxin.

In our acute toxicity experiments, 0.4 μg ^His^Dianthin‐EGF per treatment via intraperitoneal administration resulted in no biological effect and no statistically significant weight loss. Histological analyses showed no organ toxicity at this dose. Increasing the dose to 4 μg caused a significant weight loss but no adverse effects. Further increase to 40 μg caused moderate alterations in the liver by necrosis of groups of hepatocytes. The liver is the main organ that is affected when immunoconjugates are administered intraperitoneally (Ito *et al*., 1991). As we only observed moderate liver toxicity at 40 μg, it can be most likely assumed that the treatment dose of 0.35 μg, less than a hundredth of a moderately toxic dose, particularly when applied subcutaneously, is much below the highest nonobserved adverse effect level. Moreover, it can be assumed that multiple treatments will not result in an accumulation effect as the endosomal escape enhancer is almost completely excreted after 4 h (Bachran *et al*., 2010a).

The combination of ^His^Dianthin‐EGF and SO1861 revealed a very strong synergistic effect when administered subcutaneously as SO1861 alone has no toxic effect at the applied concentration and the monotherapy with ^His^Dianthin‐EGF has a 13‐fold weaker effect with respect to tumor reduction than the combination. Earlier experiments showed that intraperitoneal injection of a targeted toxin did not cause a significant decrease in tumor size when the endosomal escape enhancer is applied at the same route (Bachran *et al*., 2009). The reason for this appears to be the high concentrations of targeted toxins and enhancer at the same site resulting in inflammation and possibly toxin degradation. To avoid inflammation, SO1861 was administered s.c. at the back of the neck and ^His^Dianthin‐EGF s.c. at the vicinity of the tumor. This strategy was already successful in the past (Gilabert‐Oriol *et al*., 2013c; von Mallinckrodt *et al*., 2014); however, it required careful choice of the length of the time period between SO1861 and toxin application (Bachran *et al*., 2010a). With respect to clinical trials, the use of a deimmunized form of dianthin might facilitate systemic applications as shown for a deimmunized form of another ribosome‐inactivating protein, bouganin, in an immunotoxin with trastuzumab (Dillon *et al*., 2016). Another possibility is to substantially decrease the systemic concentration of SO1861 by modifying the enhancer to a targeted molecule.

Evaluation of the complete blood count revealed a significant change in the platelet‐derived parameters (Fig. 8). In our study, we observed that augmentation in the efficacy of treatment (combination therapy > monotherapy > placebo > untreated) led to an unexpectedly increased platelet count. This was also seen in more than a half of advanced non‐small‐cell lung cancer patients at phase II clinical trials with a combination of gemcitabine and cisplatin (Zwitter *et al*., 2005), in studies performed with a combination of gemcitabine and vincristine (Zwitter *et al*., 2001), and in pancreatic cancer patients treated with gemcitabine alone (Świeboda‐Sadlej *et al*., 2012). Thus, an increase in platelet count appears to be not uncommon, but we do not know the molecular background behind this.

Our immunohistochemical studies on tumors from xenografts showed high EGFR expression in all pancreatic tumors of untreated animals. This overexpression of EGFR in pancreatic cancer cells had also been studied by Korc *et al*. (1992). The reason for increased expression can be associated with either structural or numerical alterations of chromosome 7 (Korc *et al*., 1986; Shimizu *et al*., 1984). Over the decades, many bacterial‐ and plant‐based targeted toxins have been developed with the goal of targeting cancers reliant upon EGFR overexpression (Simon and FitzGerald, 2016). The targeted toxin 425(scFv)‐ETA, consisting of an anti‐EGFR single‐chain Fv antibody fused to a truncated *Pseudomonas* exotoxin, was strongly cytotoxic toward metastatic pancreatic cancer cells (L3.6pl) with an IC_50_ of 0.1 nm (Bruell *et al*., 2003). In mice injected with L3.6pl cells, multiple applications of 425(scFv)‐ETA reduced the number of lung metastases from 56 per mouse to 0.28 per mouse (Bruell *et al*., 2005), indicating the strong potential of this targeted toxin. Similar targeted toxins using single‐chain Fv antibodies derived from cetuximab and panitumumab resulted in IC_50_ values of 0.29 and 0.26 nm, respectively, on L3.6pl cells (Niesen *et al*., 2015). In contrast to dianthin, the truncated *Pseudomonas* exotoxin possesses its own natural translocation domain, which makes it independent from endosomal escape enhancers, explaining the similar IC_50_ values observed for the exotoxin and dianthin in the presence of SO1861.

Notably, we observed weak staining signals in tumors treated with the targeted toxin alone in comparison with placebo (Fig. 7). At least for some cancers, EGFR is a strong prognostic indicator associated with more aggressive disease and reduced survival (Oliveira‐Cunha *et al*., 2011). Therefore, on the one hand, the weak expression after ^His^Dianthin‐EGF treatment can indicate that the tumor is now less aggressive after eradicating the part of tumor cells with high expression level. On the other hand, this let us assume that a monotherapy with ^His^Dianthin‐EGF is not sufficient to kill tumor cells with lower EGFR expression resulting in selective survival of these cells and continuous tumor growth (Fig. 5). Another possibility is an adaptive decrease in EGFR expression in affected cells to develop resistance. The reason for the missing potential of ^His^Dianthin‐EGF is most likely that only a small portion of the bound and internalized targeted toxin is able to escape from the endosomes before degradation or recycling. This can be overcome in general by the use of endosomal escape enhancers (Fuchs *et al*., 2016) and for ribosome‐inactivating proteins in particular by certain glycosylated triterpenoids such as SO1861 (Bachran *et al*., 2011; Weng *et al*., 2012). The present study indicates that, in the presence of SO1861, tumor cells with low EGFR expression are also eliminated, finally leading to complete remission in four of five cases without adverse events. Thus, combining a targeted toxin with SO1861 is proven to be a very promising approach for pancreatic cancer treatment. Long‐term efficacy in mice for 60 days or more must still be investigated.

## Author contributions

HD performed all histological and immunohistochemical analyses. AW isolated and characterized SO1861 and analyzed the corresponding data. CB conducted and analyzed all other experiments and wrote the draft of the manuscript. XZ assisted in all animal experiments. MFM gave advice in glycosylated triterpenes and corrected the manuscript. HF designed and supervised the entire study, interpreted the results, and finalized the manuscript. All authors proofread the manuscript.

## Supporting information


**Fig. S1.** Western blot of ^His^Dianthin‐EGF after final purification.

**Fig. S2.** Determination of a safe SO1861 concentration.

**Fig. S3.** Images displaying dose dependent discernible physical traits of mice injected with ^His^Dianthin‐EGF.

**Fig. S4.** Densitometry scans after separation of the samples by high‐performance thin‐layer chromatography silica gel plates.

**Fig. S5.** Western blot of EGFR expression level in all applied cell lines.

**Fig. S6.** Real‐time cell analysis showing the dose‐dependent increase in cytotoxicity in BxPC‐3 cells caused by SO1861 alone.

**Table S1.** Definition of adverse effect symptoms in toxicity studies.

## References

[mol212115-bib-0001] Ali S , El‐Rayes BF , Sarkar FH and Philip PA (2005) Simultaneous targeting of the epidermal growth factor receptor and cyclooxygenase‐2 pathways for pancreatic cancer therapy. Mol Cancer Ther 4, 1943–1951. 16373709 10.1158/1535-7163.MCT-05-0065

[mol212115-bib-0002] Bachran C , Durkop H , Sutherland M , Bachran D , Muller C , Weng A , Melzig MF and Fuchs H (2009) Inhibition of tumor growth by targeted toxins in mice is dramatically improved by saponinum album in a synergistic way. J Immunother 32, 713–725. 19561537 10.1097/CJI.0b013e3181ad4052

[mol212115-bib-0003] Bachran D , Schneider S , Bachran C , Urban R , Weng A , Melzig MF , Hoffmann C , Kaufmann AM and Fuchs H (2010b) Epidermal growth factor receptor expression affects the efficacy of the combined application of saponin and a targeted toxin on human cervical carcinoma cells. Int J Cancer 127, 1453–1461. 20020492 10.1002/ijc.25123

[mol212115-bib-0004] Bachran D , Schneider S , Bachran C , Weng A , Melzig MF and Fuchs H (2011) The endocytic uptake pathways of targeted toxins are influenced by synergistically acting Gypsophila saponins. Mol Pharm 8, 2262–2272. 21981719 10.1021/mp200130j

[mol212115-bib-0005] Bachran C , Weng A , Bachran D , Riese SB , Schellmann N , Melzig MF and Fuchs H (2010a) The distribution of saponins in vivo affects their synergy with chimeric toxins against tumours expressing human epidermal growth factor receptors in mice. Br J Pharmacol 159, 345–352. 20015087 10.1111/j.1476-5381.2009.00543.x PMC2825356

[mol212115-bib-0006] Barker CJ , Leibiger IB and Berggren PO (2013) The pancreatic islet as a signaling hub. Adv Biol Regul 53, 156–163. 23073565 10.1016/j.jbior.2012.09.011

[mol212115-bib-0007] Bruell D , Bruns CJ , Yezhelyev M , Huhn M , Muller J , Ischenko I , Fischer R , Finnern R , Jauch KW and Barth S (2005) Recombinant anti‐EGFR immunotoxin 425(scFv)‐ETA’ demonstrates anti‐tumor activity against disseminated human pancreatic cancer in nude mice. Int J Mol Med 15, 305–313. 15647848

[mol212115-bib-0008] Bruell D , Stocker M , Huhn M , Redding N , Kupper M , Schumacher P , Paetz A , Bruns CJ , Haisma HJ , Fischer R *et al* (2003) The recombinant anti‐EGF receptor immunotoxin 425(scFv)‐ETA’ suppresses growth of a highly metastatic pancreatic carcinoma cell line. Int J Oncol 23, 1179–1186. 12964002 10.3892/ijo.23.4.1179

[mol212115-bib-0009] Chaga G , Hopp J and Nelson P (1999) Immobilized metal ion affinity chromatography on Co^2+^‐carboxymethylaspartate‐agarose Superflow, as demonstrated by one‐step purification of lactate dehydrogenase from chicken breast muscle. Biotechnol Appl Biochem 29(Pt 1), 19–24. 9889081

[mol212115-bib-0010] Chang CH , Gupta P , Michel R , Loo M , Wang Y , Cardillo TM and Goldenberg DM (2010) Ranpirnase (frog RNase) targeted with a humanized, internalizing, anti‐Trop‐2 antibody has potent cytotoxicity against diverse epithelial cancer cells. Mol Cancer Ther 9, 2276–2286. 20663928 10.1158/1535-7163.MCT-10-0338

[mol212115-bib-0011] Conroy T , Desseigne F , Ychou M , Bouche O , Guimbaud R , Becouarn Y , Adenis A , Raoul JL , Gourgou‐Bourgade S , de la Fouchardiere C *et al* (2011) FOLFIRINOX versus gemcitabine for metastatic pancreatic cancer. N Engl J Med 364, 1817–1825. 21561347 10.1056/NEJMoa1011923

[mol212115-bib-0012] Dillon RL , Chooniedass S , Premsukh A , Adams GP , Entwistle J , MacDonald GC and Cizeau J (2016) Trastuzumab‐deBouganin conjugate overcomes multiple mechanisms of T‐DM1 drug resistance. J Immunother 39, 117–126. 26938945 10.1097/CJI.0000000000000115

[mol212115-bib-0013] Dodson LF , Hawkins WG and Goedegebuure P (2011) Potential targets for pancreatic cancer immunotherapeutics. Immunotherapy 3, 517–537. 21463193 10.2217/imt.11.10 PMC3148788

[mol212115-bib-0014] Eser S , Schnieke A , Schneider G and Saur D (2014) Oncogenic KRAS signalling in pancreatic cancer. Br J Cancer 111, 817–822. 24755884 10.1038/bjc.2014.215 PMC4150259

[mol212115-bib-0015] Fuchs H , Weng A and Gilabert‐Oriol R (2016) Augmenting the efficacy of immunotoxins and other targeted protein toxins by endosomal escape enhancers. Toxins 8, 200. 10.3390/toxins8070200 PMC4963833 27376327

[mol212115-bib-0016] Garrido‐Laguna I and Hidalgo M (2015) Pancreatic cancer: from state‐of‐the‐art treatments to promising novel therapies. Nat Rev Clin Oncol 12, 319–334. 25824606 10.1038/nrclinonc.2015.53

[mol212115-bib-0017] Ghetie V and Vitetta ES (2001) Chemical construction of immunotoxins. Mol Biotechnol 18, 251–268. 11503519 10.1385/MB:18:3:251

[mol212115-bib-0018] Gilabert‐Oriol R , Mergel K , Thakur M , von Mallinckrodt B , Melzig MF , Fuchs H and Weng A (2013a) Real‐time analysis of membrane permeabilizing effects of oleanane saponins. Bioorg Med Chem 21, 2387–2395. 23454223 10.1016/j.bmc.2013.01.061

[mol212115-bib-0019] Gilabert‐Oriol R , Thakur M , von Mallinckrodt B , Hug T , Wiesner B , Eichhorst J , Melzig MF , Fuchs H and Weng A (2013b) Modified trastuzumab and cetuximab mediate efficient toxin delivery while retaining antibody‐dependent cell‐mediated cytotoxicity in target cells. Mol Pharm 10, 4347–4357. 24050452 10.1021/mp400444q

[mol212115-bib-0020] Gilabert‐Oriol R , Thakur M , Weise C , Dernedde J , von Mallinckrodt B , Fuchs H and Weng A (2013c) Small structural differences of targeted anti‐tumor toxins result in strong variation of protein expression. Protein Expr Purif 91, 54–60. 23867360 10.1016/j.pep.2013.07.004

[mol212115-bib-0021] Gilabert‐Oriol R , Weng A , Trautner A , Weise C , Schmid D , Bhargava C , Niesler N , Wookey PJ , Fuchs H and Thakur M (2015) Combinatorial approach to increase efficacy of Cetuximab, Panitumumab and Trastuzumab by dianthin conjugation and co‐application of SO1861. Biochem Pharmacol 97, 247–255. 26253687 10.1016/j.bcp.2015.07.040

[mol212115-bib-0022] Grisshammer R and Tucker J (1997) Quantitative evaluation of neurotensin receptor purification by immobilized metal affinity chromatography. Protein Expr Purif 11, 53–60. 9325139 10.1006/prep.1997.0766

[mol212115-bib-0023] Heisler I , Sutherland M , Bachran C , Hebestreit P , Schnitger A , Melzig MF and Fuchs H (2005) Combined application of saponin and chimeric toxins drastically enhances the targeted cytotoxicity on tumor cells. J Control Release 106, 123–137. 15935506 10.1016/j.jconrel.2005.04.006

[mol212115-bib-0024] Hochuli E , Dobeli H and Schacher A (1987) New metal chelate adsorbent selective for proteins and peptides containing neighbouring histidine residues. J Chromatogr 411, 177–184. 3443622 10.1016/s0021-9673(00)93969-4

[mol212115-bib-0025] Huhn M , Sasse S , Tur MK , Matthey B , Schinköthe T , Rybak SM , Barth S and Engert A (2001) Human angiogenin fused to human CD30 ligand (Ang‐CD30L) exhibits specific cytotoxicity against CD30‐positive lymphoma. Cancer Res 61, 8737. 11751393

[mol212115-bib-0026] Ioannou N , Dalgleish AG , Seddon AM , Mackintosh D , Guertler U , Solca F and Modjtahedi H (2011) Anti‐tumour activity of afatinib, an irreversible ErbB family blocker, in human pancreatic tumour cells. Br J Cancer 105, 1554–1562. 21970876 10.1038/bjc.2011.396 PMC3242519

[mol212115-bib-0027] Ito T , Qiu H , Collins JA , Brill AB , Johnson DK and Griffin TW (1991) Preclinical assessments of 90Y‐labeled C110 anti‐carcinoembryonic antigen immunotoxin: a therapeutic immunoconjugate for human colon cancer. Cancer Res 51, 255–260. 1988087

[mol212115-bib-0028] Jones S , Zhang X , Parsons DW , Lin JC‐H , Leary RJ , Angenendt P , Mankoo P , Carter H , Kamiyama H , Jimeno A *et al* (2008) Core signaling pathways in human pancreatic cancers revealed by global genomic analyses. Science 321, 1801–1806. 18772397 10.1126/science.1164368 PMC2848990

[mol212115-bib-0029] Kleeff J , Michalski C , Friess H and Buchler MW (2006) Pancreatic cancer: from bench to 5‐year survival. Pancreas 33, 111–118. 16868475 10.1097/01.mpa.0000229010.62538.f2

[mol212115-bib-0030] Korc M , Chandrasekar B , Yamanaka Y , Friess H , Buchier M and Beger HG (1992) Overexpression of the epidermal growth factor receptor in human pancreatic cancer is associated with concomitant increases in the levels of epidermal growth factor and transforming growth factor alpha. J Clin Invest 90, 1352–1360. 1401070 10.1172/JCI116001 PMC443180

[mol212115-bib-0031] Korc M , Meltzer P and Trent J (1986) Enhanced expression of epidermal growth factor receptor correlates with alterations of chromosome 7 in human pancreatic cancer. Proc Natl Acad Sci U S A 83, 5141–5144. 3014534 10.1073/pnas.83.14.5141 PMC323906

[mol212115-bib-0032] von Mallinckrodt B , Thakur M , Weng A , Gilabert‐Oriol R , Durkop H , Brenner W , Lukas M , Beindorff N , Melzig MF and Fuchs H (2014) Dianthin‐EGF is an effective tumor targeted toxin in combination with saponins in a xenograft model for colon carcinoma. Future Oncol 10, 2161–2175. 25471031 10.2217/fon.14.164

[mol212115-bib-0033] Moore MJ , Goldstein D , Hamm J , Figer A , Hecht JR , Gallinger S , Au HJ , Murawa P , Walde D , Wolff RA *et al* (2007) Erlotinib plus gemcitabine compared with gemcitabine alone in patients with advanced pancreatic cancer: a phase III trial of the National Cancer Institute of Canada Clinical Trials Group. J Clin Oncol 25, 1960–1966. 17452677 10.1200/JCO.2006.07.9525

[mol212115-bib-0034] Morris JP , Wang SC , Hebrok M (2010) KRAS, Hedgehog, Wnt and the twisted developmental biology of pancreatic ductal adenocarcinoma. Nat Rev Cancer 10, 683–695. 20814421 10.1038/nrc2899 PMC4085546

[mol212115-bib-0035] Niesen J , Stein C , Brehm H , Hehmann‐Titt G , Fendel R , Melmer G , Fischer R and Barth S (2015) Novel EGFR‐specific immunotoxins based on panitumumab and cetuximab show in vitro and ex vivo activity against different tumor entities. J Cancer Res Clin Oncol 141, 2079–2095. 25899161 10.1007/s00432-015-1975-5 PMC11824141

[mol212115-bib-0036] Oliveira‐Cunha M , Newman WG and Siriwardena AK (2011) Epidermal growth factor receptor in pancreatic cancer. Cancers 3, 1513. 24212772 10.3390/cancers3021513 PMC3757375

[mol212115-bib-0037] Pepys MB , Hawkins PN , Kahan MC , Tennent GA , Gallimore JR , Graham D , Sabin CA , Zychlinsky A and de Diego J (2005) Pro‐inflammatory effects of bacterial recombinant human C‐reactive protein are caused by contamination with bacterial products not by C‐reactive protein itself. Circ Res 97, e97–e103. 16254214 10.1161/01.RES.0000193595.03608.08 PMC1400607

[mol212115-bib-0038] Pietrantonio F , Perrone F , Biondani P , Maggi C , Lampis A , Bertan C , Venturini F , Tondulli L , Ferrari D , Ricci V *et al* (2013) Single agent panitumumab in KRAS wild‐type metastatic colorectal cancer patients following cetuximab‐based regimens: clinical outcome and biomarkers of efficacy. Cancer Biol Ther 14, 1098–1103. 24025413 10.4161/cbt.26343 PMC3912032

[mol212115-bib-0039] Robichon C , Luo J , Causey TB , Benner JS and Samuelson JC (2011) Engineering *Escherichia coli* BL21(DE3) derivative strains to minimize *E. coli* protein contamination after purification by immobilized metal affinity chromatography. Appl Environ Microbiol 77, 4634–4646. 21602383 10.1128/AEM.00119-11 PMC3127686

[mol212115-bib-0040] Salomon DS , Brandt R , Ciardiello F and Normanno N (1995) Epidermal growth factor‐related peptides and their receptors in human malignancies. Crit Rev Oncol Hematol 19, 183–232. 7612182 10.1016/1040-8428(94)00144-i

[mol212115-bib-0041] Shimizu N , Kondo I , Gamou S , Behzadian MA and Shimizu Y (1984) Genetic analysis of hyperproduction of epidermal growth factor receptors in human epidermoid carcinoma A431 cells. Somat Cell Mol Genet 10, 45–53. 6322359 10.1007/BF01534472

[mol212115-bib-0042] Siegel RL , Miller KD and Jemal A (2015) Cancer statistics, 2015. CA Cancer J Clin 65, 5–29. 25559415 10.3322/caac.21254

[mol212115-bib-0043] Simon N , FitzGerald D (2016) Immunotoxin therapies for the treatment of epidermal growth factor receptor‐dependent cancers. Toxins 8, 137. 10.3390/toxins8050137 PMC4885052 27153091

[mol212115-bib-0044] Strocchi P , Barbieri L and Stirpe F (1992) Immunological properties of ribosome‐inactivating proteins and a saporin immunotoxin. J Immunol Methods 155, 57–63. 1401966 10.1016/0022-1759(92)90271-t

[mol212115-bib-0045] Świeboda‐Sadlej A , Kraj L , Krawczyk J , Nita E and Dwilewicz‐Trojaczek J (2012) Thrombocytosis in patients with pancreatic cancer treated with gemcitabine – does it have clinical significance? Description of 6 cases. Contemp Oncol (Pozn) 16, 353–355. 23788909 10.5114/wo.2012.30068 PMC3687435

[mol212115-bib-0046] Von Hoff DD , Ervin T , Arena FP , Chiorean EG , Infante J , Moore M , Seay T , Tjulandin SA , Ma WW , Saleh MN *et al* (2013) Increased survival in pancreatic cancer with nab‐paclitaxel plus gemcitabine. N Engl J Med 369, 1691–1703. 24131140 10.1056/NEJMoa1304369 PMC4631139

[mol212115-bib-0047] Weng A , Thakur M , Beceren‐Braun F , Bachran D , Bachran C , Riese SB , Jenett‐Siems K , Gilabert‐Oriol R , Melzig MF and Fuchs H (2012) The toxin component of targeted anti‐tumor toxins determines their efficacy increase by saponins. Mol Oncol 6, 323–332. 22309811 10.1016/j.molonc.2012.01.004 PMC5528334

[mol212115-bib-0048] Xie D and Xie K (2015) Pancreatic cancer stromal biology and therapy. Genes Dis 2, 133–143. 26114155 10.1016/j.gendis.2015.01.002 PMC4476547

[mol212115-bib-0049] Yu L‐Y , Su G‐M , Chen C‐K , Chiang Y‐T and Lo C‐L (2016) Specific cancer cytosolic drug delivery triggered by reactive oxygen species‐responsive micelles. Biomacromol 17, 3040–3047. 10.1021/acs.biomac.6b00916 27536957

[mol212115-bib-0050] Zwitter M , Cufer T and Wein W (2001) Gemcitabine and vincristine: an effective outpatient regimen with low myelotoxicity for stage IV non‐small cell lung cancer. Neoplasma 48, 200–202. 11583289

[mol212115-bib-0051] Zwitter M , Kovac V , Smrdel U , Kocijancic I , Segedin B and Vrankar M (2005) Phase I‐II trial of low‐dose gemcitabine in prolonged infusion and cisplatin for advanced non‐small cell lung cancer. Anticancer Drugs 16, 1129–1134. 16222156 10.1097/00001813-200511000-00013

