# Supplementary figures and images for: Targeted dianthin is a powerful toxin to treat pancreatic carcinoma when applied in combination with the glycosylated triterpene SO1861
Source: Mol Oncol. 2017 Sep 15;11(11):1527–43. doi: 10.1002/1878-0261.12115 (PMC5664001; doi:10.1002/1878-0261.12115)

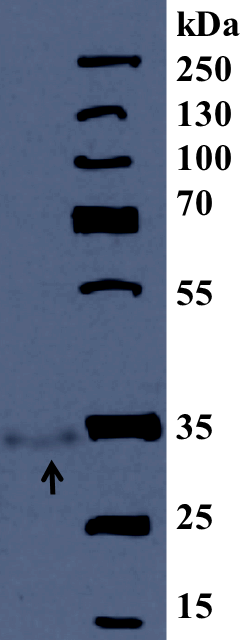

Supplement: Supplementary file 1 — Fig. S1. Western blot of HisDianthin‐EGF after final purification. [file MOL2-11-1527-s001.tif]

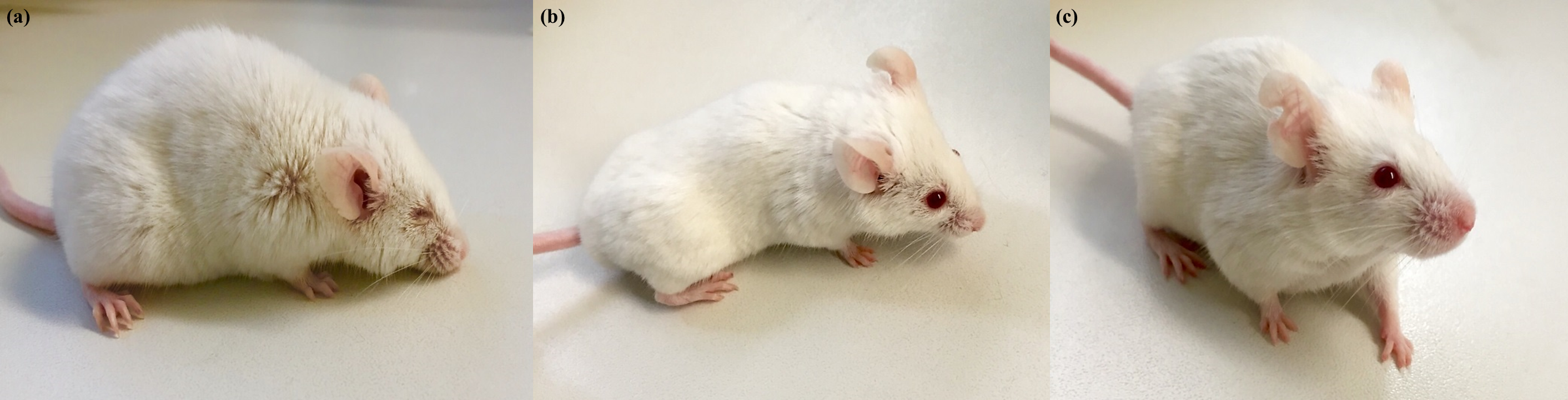

Supplement: Supplementary file 3 — Fig. S3. Images displaying dose dependent discernible physical traits of mice injected with HisDianthin‐EGF. [file MOL2-11-1527-s003.tif]

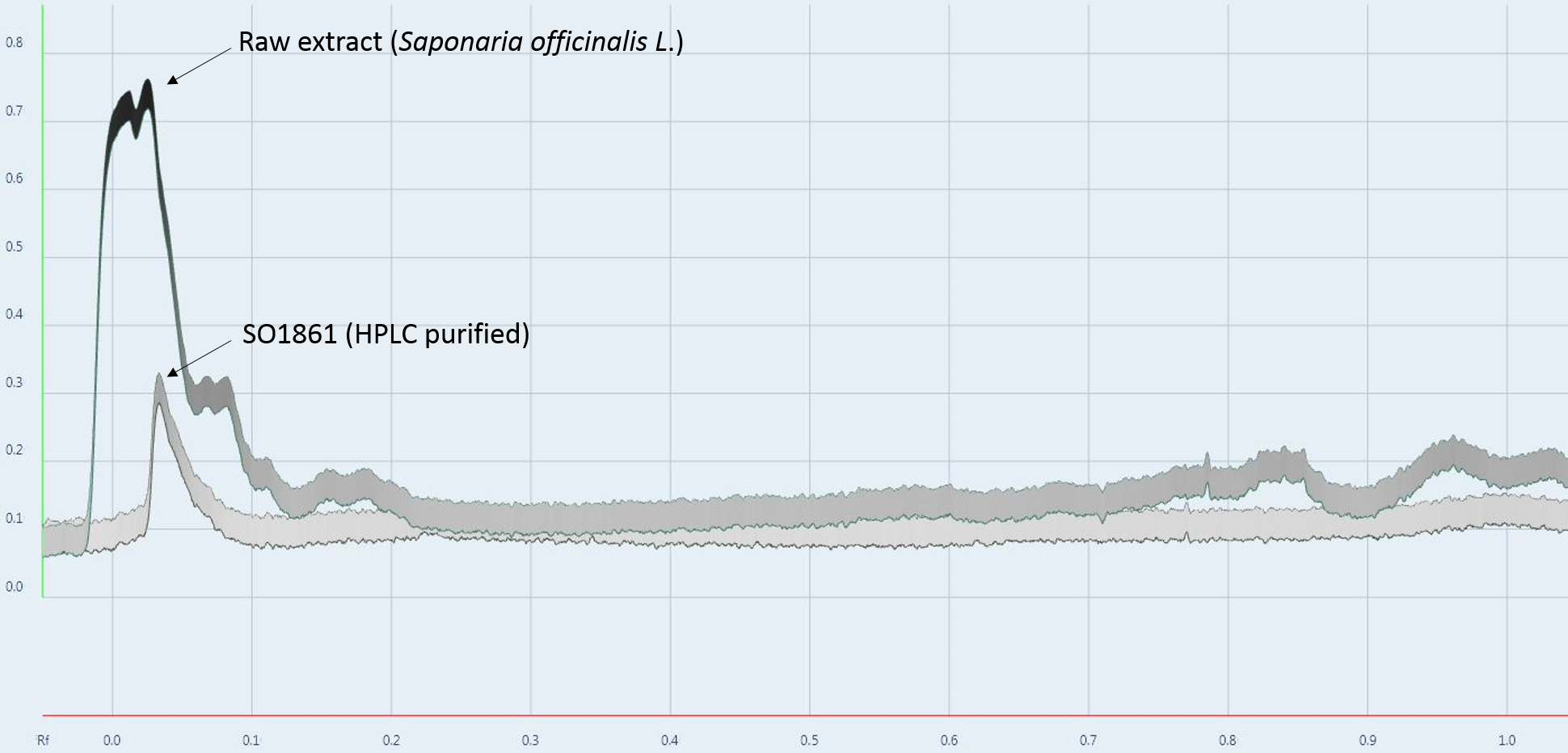

Supplement: Supplementary file 4 — Fig. S4. Densitometry scans after separation of the samples by high‐performance thin‐layer chromatography silica gel plates. [file MOL2-11-1527-s004.tif]

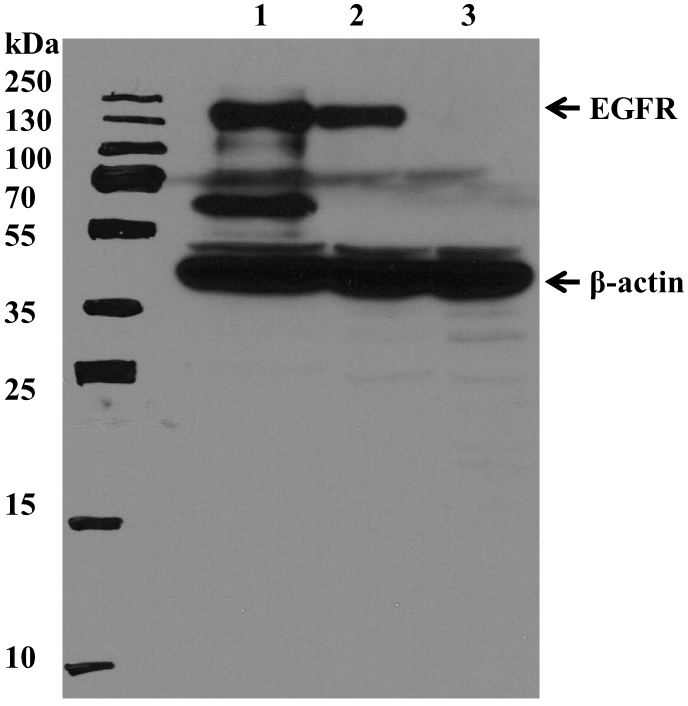

Supplement: Supplementary file 5 — Fig. S5. Western blot of EGFR expression level in all applied cell lines. [file MOL2-11-1527-s005.tif]

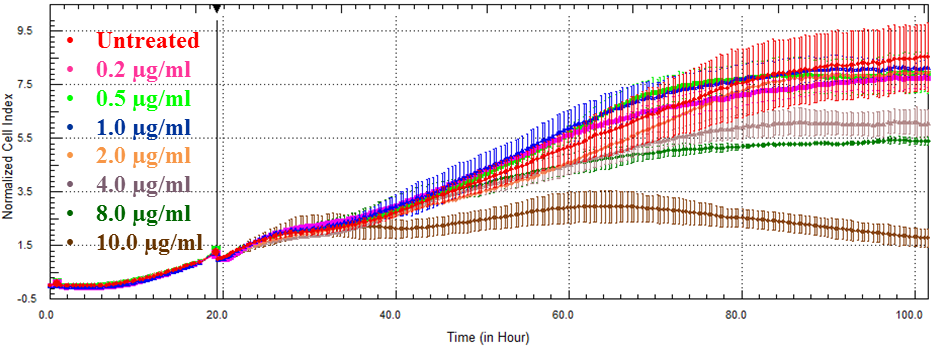

Supplement: Supplementary file 6 — Fig. S6. Real‐time cell analysis showing the dose‐dependent increase in cytotoxicity in BxPC‐3 cells caused by SO1861 alone. [file MOL2-11-1527-s006.tif]
