# Supplementary material for: Targeted dianthin is a powerful toxin to treat pancreatic carcinoma when applied in combination with the glycosylated triterpene SO1861
Source: Mol Oncol. 2017 Sep 15;11(11):1527–43. doi: 10.1002/1878-0261.12115 (PMC5664001; doi:10.1002/1878-0261.12115)
Supplement: Supplementary file 7 — Table S1. Definition of adverse effect symptoms in toxicity studies. [file MOL2-11-1527-s007.pdf]

Supplementary Table

Symptoms for toxicity are specified according to the following table that was approved by the authorities. Category A: negligible effects; category B: weak effects; category T: toxic (adverse) effects. Body condition scores (BCS): Body condition (BC) 1: Mouse is emaciated (skeletal structure extremely prominent); BC 2: Mouse is under-conditioned (segmentation of vertebral column evident).

**Treatment specific symptoms**	**Category**
Skin irritation or small areas of hair loss (≤ 1 cm^2)	A
Large areas of hair loss (> 1 cm^2)	T
Local inflammation at the injection site	B
Slightly shallow breathing	B
Tingling or apathy or markedly shallow breathing	T
**Weight and body condition scoring (BCS)**	
Body weight loss < 15% but tendency of the past 24 h increasing	A
Body weight loss < 15% and tendency of the past 24 h decreasing	B
Body weight loss ≥ 15% or BC 1 or BC 2	T
**Other symptoms**	
Shaggy fur or scratches	A
Retarded motion	A
Decreased food intake	A
Separation of the animal	A
Dehydration	B
Temporary squatting or trembling	B
Sunken or dull eyes, or lividness at eyes, ears or skin	T
Motor abnormalities such as paralysis	T
Superficial injuries (e.g. bite wounds)	T
Automutilation of limbs	T
Blood on body openings or bloody feces	T
Systemic infections	T
Constant squatting or trembling, or abnormal posture	T
Paralysis	T
Rectal prolapse	T
Dark discoloration of the abdomen	T
Moribund	T
