# Supplementary material for: Targeted dianthin is a powerful toxin to treat pancreatic carcinoma when applied in combination with the glycosylated triterpene SO1861
Source: Mol Oncol. 2017 Sep 15;11(11):1527–43. doi: 10.1002/1878-0261.12115 (PMC5664001; doi:10.1002/1878-0261.12115)
Supplement: Supplementary file 8 [file MOL2-11-1527-s008.docx]

**Supplementary Figure 1.** Western blot of HisDianthin-EGF (arrow) after final purification.

The protein was detected with an anti-His-tag monoclonal antibody conjugated to HRP

(Clontech laboratories, Inc., France).

**Supplementary Figure 2.** Determination of a safe SO1861 concentration. (a) BxPC-3, (b)

MIA PaCa-2 and (c) NIH-3T3 cells were seeded in 96-well plates and grown for 24 h. Cells

were then treated with SO1861 (0.13 to 32 μg/ml final concentration) or not (used as

reference value). Cells were further incubated for 72 h. Viability was determined by an MTT

assay.

**Supplementary Figure 3.** Images displaying dose dependent discernible physical traits of

mice injected with (a) 40 μg/mouse, (b) 4 μg/mouse and (c) 0.4 μg/mouse.

**Supplementary Figure 4.** Densitometry scans after separation of the samples by highperformance

thin-layer chromatography silica gel plates. The curves show high-performance

liquid chromatography-purified SO1861 and the raw extract, from which SO1861 was

isolated. Absorption was measured at 600 nm after derivatization with sulfuric acid (10%)

**Supplementary Figure 5:** Western blot of EGFR expression level in (1) BxPC-3, (2) MIA

PaCa-2 and (3) NIH-3T3 cells. EGFR was detected with a rabbit anti-EGFR antibody (Abcam

plc, UK). The loading control β-actin was detected with a mouse anti-β-actin antibody

(Abcam plc, UK).

Supplementary Figure 6: Real time cell analysis showing the dose-dependent increase in

cytotoxicity in BxPC-3 cells caused by SO1861 alone (0.2 to 10 μg/ml final concentration).

The y-axis shows the impedance-based cell index that was normalized after 19 h just before

treatment started. The normalized cell index can be assumed to be proportional to the number

of living cells.

**Supplementary Table 1:** Definition of adverse effect symptoms in toxicity studies
